# Factors associated with successful purchase of cigarettes among students aged 11–17 years who made a purchase attempt in The Gambia: Evidence from the 2017 Global Youth Tobacco Survey

**DOI:** 10.18332/tid/168669

**Published:** 2023-08-10

**Authors:** Bai Cham, Lucy Popova, Omar Badjie, Scott R. Weaver

**Affiliations:** 1 School of Public Health, Georgia State University, Atlanta, United States; 2 Disease Control and Elimination Theme, Medical Research Council Unit The Gambia at London School of Hygiene and Tropical Medicine, Fajara, The Gambia; 3 Non-Communicable Disease Program Unit, Ministry of Health, Banjul, The Gambia

**Keywords:** smoke-free policies, students, access, survey research

## Abstract

**INTRODUCTION:**

Access to tobacco plays a strong role in smoking initiation among youth. This study aimed to quantify successful purchase of cigarettes and assess the factors associated with cigarette purchase access among students aged 11–17 years in The Gambia.

**METHODS:**

We used the 2017 Global Youth Tobacco Survey (GYTS) of 12585 students, aged 11–17 years from The Gambia. The analysis was restricted to 2951 students aged 11–17 years who bought and/or attempted to buy cigarettes within the past 30 days regardless of smoking status. Our outcome was purchasing access, defined as successfully purchasing cigarettes within the past 30 days. We report a weighted prevalence of successful purchase attempt. Multivariable logistic regression assessed the factors associated with successful purchase of cigarettes and was adjusted for non-response and the complex survey design.

**RESULTS:**

Most students (59.4%, 95% CI: 55.4–63.3) who attempted to purchase cigarettes were successful, most of whom were boys (61.3%, 95% CI: 57.8–64.6). However, there was no significant difference in successful attempts to purchase cigarettes between boys (59.3%, 95% CI: 54.2–64.2) and girls (59.4%, 95% CI: 55.4–63.3). Older age (AOR=2.45; 95% CI: 1.25–4.78), higher school grade (AOR=1.53; 95% CI: 1.09–2.16) and current cigarettes smoking (AOR=1.41; 95% CI: 1.08–1.85) were significantly associated with successful attempt to purchase cigarettes. Sex, parents’ smoking status and students’ weekly pocket money were not associated with successful cigarettes purchase attempt. Among students who currently smoke cigarettes, 55.6% (95% CI: 47.7–63.2) bought them from a store, shop, street vendor, or canteen; 12.2% (95% CI: 8.3–17.5) obtained them from someone else, and 6.7% (95% CI: 4.4–10.0) used other means the last time they smoked.

**CONCLUSIONS:**

Students in The Gambia can purchase cigarettes without much hinderance despite the restrictions. Our research findings can guide the implementation of policies, programs, and public health strategies including more efforts toward implementing tobacco control laws and regulations that protect children from exposure and use of all forms of tobacco products.

## INTRODUCTION

More than 80% of the one billion plus global tobacco users live in low- and middle-income countries (LMICs)^1^. Most people who smoke tobacco start smoking as children/youth, and youth initiators are more likely to be active smokers in adulthood^2-4^. Access to tobacco products plays a strong role in smoking initiation among youth^2,5,6^. For example, a study of secondary school students (aged 12–20 years) in The Gambia found that nearly 40% of non-smokers were susceptible to cigarette smoking, and susceptibility to cigarette smoking was more common among students who were sent to buy cigarettes^6^. Evidence has shown that early tobacco smoking initiation predicts increased chances of nicotine dependence, longer duration of smoking, and difficulty in quitting^7,8^.

Article 16 of the World Health Organization Framework Convention on Tobacco Control (WHO FCTC) indicates that parties have an obligation to prohibit the sale of tobacco to youth^9^. The Gambia became a party to the WHO FCTC in 2005 and ratified it in September 2007^10^. The Ministry of Health of The Gambia has implemented a tobacco control Act and numerous policies, some of which prohibit the sale of tobacco to and by minors (aged <18 years)^11^. The Gambia has enacted several significant regulations and policies on tobacco control, including the Smoking (Prohibition in Public Places) Act 1998^12^, the Tobacco Control Act 2016^13^, and the Tobacco Control Regulations 2019^14^. Based on international treaties and local regulations, the country has the legal obligation and instruments to protect minors from exposure to tobacco. Although the Tobacco Control Act, 2016, was adopted in 2018, a year after this survey, some of the provisions, including those prohibiting sales to and by minors, came into effect immediately in 2016^13^.

Despite the numerous policy achievements in tobacco control, there remain gaps, especially regarding the implementation of regulations that protect minors. Studies in The Gambia found that 26% of boys and 9% of girls reported ever having smoked cigarettes^15^, while a subnational Global Youth Tobacco Survey conducted among youths aged 13–15 years in the capital of Banjul reported a prevalence of ever cigarette smoking among boys and girls of 29% and 20%, respectively^16^. The GYTS 2017 factsheet reported 9.2% of students aged 13–15 years currently smoke cigarettes^17^. A survey among adolescents and adults aged 15–64 years in The Gambia have shown that 66.1% were exposed to secondhand smoke in public places and the exposure levels were higher in males (79.9%) than females (58.7%)^18^.

Whereas the evidence has shown that Gambian children have access to tobacco products^6,15,19^, there have been no studies on purchase attempts and the factors associated with successful purchase attempts of cigarettes among students in The Gambia. Studies conducted in six other Sub-Saharan African countries including Ghana, Ivory Coast, Swaziland, Uganda and Republic of Congo^20^ , and other studies conducted in Brazil^21^, Vietnam^22^, and China^23^ have also shown a high proportion of students were successfully purchasing cigarettes. To develop stronger policies to address this issue, it is important to assess the contributing factors associated with students’ access to tobacco. This study aimed to quantify successful purchase of cigarettes and assess the factors associated with successful attempt to purchase cigarettes among students aged 11–17 years in The Gambia.

## METHODS

We used the 2017 Global Youth Tobacco Survey (GYTS) of 12585 students, aged 11–17 years, attending school grades 7–9 in The Gambia. Two GYTS surveys have been conducted in The Gambia. The first one was a subnational survey conducted in the capital city Banjul in 2008^16^, while the second and most recent was conducted in 2017 and is nationally representative of youths in the country with a school response rate of 100% (145/145 schools) and an individual interview response rate of 86.5%^17^. Details of The Gambia 2017 survey methodology have been published elsewhere^24^. In brief, GYTS is designed by the US Centers for Disease Control and Prevention and collaborating partners and the sample is drawn using a two-stage cluster-sampling design^25,26^. Schools are selected with probability proportional to size during the first stage, and then classes within participating schools are selected as a systematic probability sampling in the second stage^27^. Our analysis was restricted to 2951 students aged 11–17 years who bought and/or attempted to buy cigarettes within the past 30 days.

### Outcome and predictor variables

The main outcome variable was cigarette purchasing access and was defined as successfully purchasing cigarettes within the past 30 days regardless of smoking status, assessed with the question: ‘During the past 30 days, did anyone refuse to sell you cigarettes because of your age?’ with response options ‘I did not try to buy cigarettes during the past 30 days’, ‘Yes, someone refused to sell me cigarettes because of my age’, and ‘No, my age did not keep me from buying cigarettes’. Those who did not try to buy cigarettes within the past 30 days were excluded from the analysis. To determine how students who currently smoke cigarettes obtained them, we used the question: ‘The last time you smoked cigarettes during the past 30 days, how did you get them?’. The responses included: from a retail outlet (including store or shop, street vendor, canteen, vending machine kiosk), from someone else, or through other means; but participants could select only one answer. The questions in the GYTS survey questionnaire are validated and other studies including one that used GYTS data from 140 countries used the above definitions^26,28,29^.

Predictor variables include age, sex, school grade, average weekly pocket money, current smoking, and parents’ smoking status. Current smoking was defined as having smoked cigarettes within the past 30 days, assessed using the question: ‘During the past 30 days, on how many days did you smoke cigarette?’^29^. Whether youths had ever tried or experimented with cigarettes was assessed using the question: ‘Have you ever tried or experimented with cigarette smoking, even one or two puffs?’^29^. For ease of analysis, we combined the two variables on cigarette smoking into a single variable with three categories including: ‘never smoked cigarettes’; ‘ever tried cigarette smoking, but not currently smoking’; and ‘currently smoke cigarettes’.

### Statistical analysis

Our analyses accounted for the complex survey design by incorporating sampling weights and the stratification and cluster variables using the *svy* command in Stata (version 17). This was done to adjust for differences between the age–sex distribution of the target population and that of the achieved sample to ensure the sample was nationally representative of students aged 11–17 years. We calculated weighted prevalence (with corresponding 95% confidence intervals) of successful cigarette purchase by sociodemographics, to cigarettes by sociodemographic and other variables of interest.

Chi-squared statistical tests of association were conducted to evaluate the association. Multivariable logistic regression assessed the factors associated with successful purchase of cigarettes among children aged 11–17 years. All statistical tests were two-tailed with α=0.05. All analysis were conducted using Stata version 17.

## RESULTS

Table 1 summarizes the sociodemographic characteristics of students who had made a past 30 days cigarette purchase attempt in our sample. More than half of the participants were boys (61.2%, 95% CI: 57.8–64.6) and 66.8% (95% CI: 63.5–70.0) of the parents of the children were non-smokers. Most of the students were in grade 7 (37.5%, 95% CI: 30.7–44.8) and grade 8 (36.2%, 95%: CI: 30.5–42.3) and only 26.3% (95% CI: 20.3–33.4) were in grade 9 (Table 1). Most of the students who attempted to purchase cigarettes (57.9%, 95% CI: 53.2–62.5%) never smoked, 19.5% (95% CI: 16.8–22.4) had ever tried cigarette smoking but not currently smoking, and 22.6% (95% CI: 19.8–25.7) currently smoked cigarettes.

**Table 1:** Characteristics of study participants aged 11–17 years from The Gambia GYTS 2017 Cross-Sectional Survey (N=2951)

*Characteristics*	Unweighted frequency*	*Weighted% (95% CI)*
**Sex**		
Male	1576	61.2 (57.8–64.6)
Female	1311	38.8 (35.4–42.2)
**Age** (years)		
11	116	2.6 (2.0–3.5)
12	204	5.9 (0.6–7.2)
13	416	13.1 (11.0–15.6)
14	636	22.8 (20.6–25.2)
15	560	19.0 (16.9–21.2)
16	454	16.5 (14.1–19.2)
17	459	20.2 (17.1–23.6)
**Grade**		
7	1163	37.5 (30.7–44.8)
8	1064	36.2 (30.5–42.3)
9	690	26.3 (20.3–33.4)
**Students’ average weekly income** (GMD)		
Usually don’t have any money	346	10.1 (8.4–12.0)
<25	1058	31.7 (29.0–34.4)
25–50	925	33.9 (31.1–36.8)
51–100	302	12.5 (10.7–14.6)
101–150	114	4.9 (3.7–6.3)
151–200	64	2.8 (1.9–4.1)
>200	105	4.2 (3.4–5.1)
**Cigarette smoking status**		
Never smoked cigarettes	1673	57.9 (53.2–62.5)
Ever tried cigarette smoking, but not currently smoking	497	19.5 (16.8–22.4)
Currently smoke cigarettes	492	22.6 (19.8–25.7)
**Parent’s cigarette smoking status**		
Neither	1916	66.8 (63.5–70.0)
Both	169	4.5 (3.6–5.7)
Father only	420	15.8 (13.4–18.6)
Mother only	188	5.7 (4.5–7.2)
Don’t know	229	7.1 (5.7–8.9)

* Some of the frequencies do not add up to 2951 because of missing variables. GMD: 1000 Gambian Dalasi about US$22 (2017).

**Figure 1:**
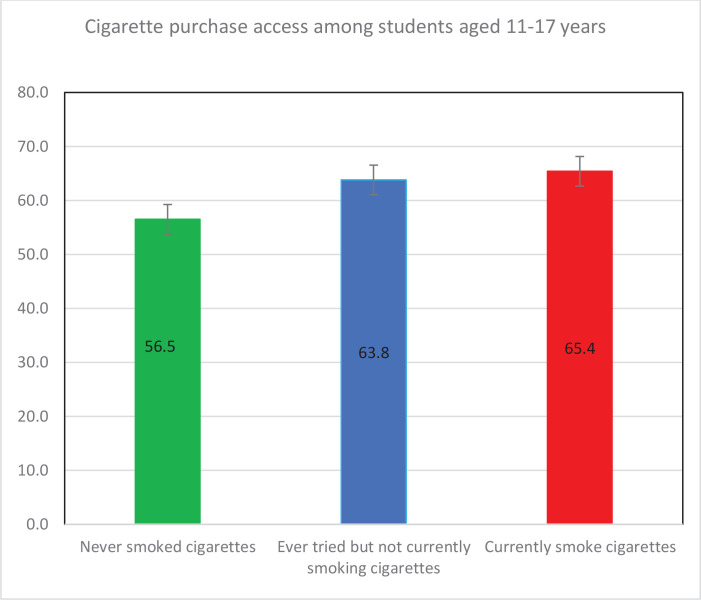
Cigarette purchase access by smoking status among students aged 11–17 years from The Gambia 2017 Cross-Sectional GYTS Survey (N=2951)

A high proportion of students purchased cigarettes in the past 30 days (59.4%, 95% CI: 55.4–63.3) and were not refused purchase because of their age (Table 2). This was higher among students who currently smoked cigarettes (65.4%, 95% CI: 59.7–70.7) or ever tried cigarette smoking, but not currently smoking (63.8%, 95%CI: 57.0–70.0) compared with those who never smoke cigarettes (56.5%, 95% CI: 51.9–61.1) (Figure 1). Most of the students who attempted to purchase cigarettes were boys (61.2% vs 38.8%) (Table 1), but there was no significant difference in successful attempt to purchase cigarettes between boys (59.3%, 95% CI: 54.2–64.2) and girls (59.4%, 95% CI: 55.4–63.3) (Table 2). Successful purchase of cigarettes significantly increased with age and school grade. It was also significantly higher among students who currently smoked compared with those who did not. On how they accessed the cigarettes they most recently smoked, 55.6% (95% CI: 47.7–63.2) of students who currently smoked cigarettes bought them from a store, shop, street vendor, or canteen, 12.2% (95% CI: 8.3–17.5) obtained them from someone else, and 6.7% (95% CI: 4.4–10.0) used other means.

**Table 2:** Prevalence and factors associated with successful cigarette purchase attempt among students aged 11–17 years from The Gambia GYTS 2017 Cross-Sectional Survey (N=2951)

	*Prevalence of access (successful purchase attempt) % (95% CI)*		*Factors associated with successful attempt to purchase cigarettes*	
		*p*	*OR (95% CI)*	*AOR (95% CI)*
**All**	59.4 (55.4–63.3)			
**Sex**		0.908		
Male (Ref.)	59.3 (54.2–64.2)		1	1
Female	59.6 (55.1–63.9)		1.01 (0.81–1.27)	1.14 (0.88–1.49)
**Age** (years)		0.001		
11 (Ref.)	42.4 (31.0–54.8)		1	1
12	45.3 (36.4–54.6)		1.13 (0.62–2.04)	1.15 (0.57–2.34)
13	57.2 (50.2–64.0)		1.81 (1.05–3.12) *	2.06 (1.06–3.99) *
14	56.7 (49.5–63.7)		1.78 (1.07–2.95) *	1.89 (0.99–3.59)
15	61.2 (59.0–66.3)		2.14 (1.20–3.81) **	1.89 (0.96–3.71)
16	56.7 (49.3–63.9)		1.78 (1.04–3.04) *	1.53 (0.81–2.86)
17	68.8 (60.8–75.8)		2.99 (1.77–5.04) ***	2.45 (1.25–4.78) **
**Grade**		0.006		
7 (Ref.)	53.0 (48.4–57.7)		1	1
8	61.0 (54.3–67.4)		1.39 (1.01–1.90) *	1.38 (0.99–1.92) *
9	65.6 (59.2–71.5)		1.69 (1.27–2.25) ***	1.53 (1.09–2.16) **
**Students’ average weekly income** (GMD)		0.234		
Usually don’t have any money (Ref.)	59.5 (53.9–64.9)		1	1
<25	59.5 (53.8–64.9)		1.00 (0.74–1.35)	0.83 (0.59–1.16)
25–50	57.9 (53.6–65.0)		0.94 (0.71–1.23)	0.75 (0.53–1.04)
51–100	54.1 (45.7–62.3)		0.80 (0.55–1.16)	0.59 (0.38–0.91) *
101–150	70.1 (57.8–80.0)		1.59 (0.91–2.78)	1.30 (0.73–2.33)
151–200	62.2 (53.9–81.2)		1.53 (0.81–2.88)	1.63 (0.83–3.17)
>200	62.0 (45.6–76.1)		1.11 (0.56–2.21)	0.96 (0.43–2.15)
**Cigarette smoking status**		0.010		
Never smoked cigarettes (Ref.)	56.5 (51.9–61.1)		1	1
Ever tried cigarette smoking, but not currently smoking	63.8 (57.0–70.0)		1.35 (1.00–1.81) *	1.34 (0.95–1.91)
Currently smoke cigarettes	65.4 (59.7–70.7)		1.45 (1.10–1.92) **	1.41 (1.08–1.85) **
**Parent’s cigarette smoking status**		0.652		
Neither (Ref.)	58.1 (53.5–62.6)		1	1
Both	62.8 (54.6–70.3)		1.22 (0.83–1.79)	0.99 (0.63–1.51)
Father only	62.2 (55.9–68.1)		1.18 (0.91–1.54)	0.99 (0.73–1.34)
Mother only	60.6 (47.8–72.0)		1.11 (0.69–1.78)	0.91 (0.58–1.42)
Don’t know	60.8 (53.3–67.8)		1.11 (0.78–1.58)	1.00 (0.71–1.42)

AOR: adjusted odds ratio.

* p<0.05,

** p≤0.01,

*** p≤0.001; p-value for χ^2^ test.

GMD: 1000 Gambian Dalasi about US$22 (2017).

### Factors associated with successful purchase of cigarettes

In our fully adjusted models, older age (AOR=2.45; 95% CI: 1.25–4.78), higher school grade (AOR=1.53; 95% CI: 1.09–2.16) and current cigarettes smoking (AOR=1.41; 95% CI: 1.08–1.85) were significantly associated with a successful attempt to purchase cigarettes (Table 2). Students’ sex, parents’ smoking status and students’ weekly pocket money were not associated with successful cigarettes purchase attempt.

## DISCUSSION

Our findings indicate that most of the students (59.4%) in The Gambia who attempted to purchase cigarettes were not prevented from doing so because of their age. A high proportion of the students who attempted to purchase cigarettes were non-smokers (76.5%), and 58.5% of them were successful in purchasing cigarettes, resulting in exposing youth, who are not currently using cigarettes, to the products. Easy access to cigarettes among students who do not use tobacco as a result of lack of laws or weak implementation of regulations that protect children where they exist may lead to subsequent cigarette smoking initiation and addiction to tobacco^30,31^. Research evidence has shown that non-smoking students aged 12–20 years in The Gambia who were sent to buy cigarettes were more susceptible to initiation of tobacco smoking than those who did not have to buy cigarettes^6^.

Most of the previous studies that assessed youths’ purchase access to cigarettes focused on youth who were current cigarette smokers. These include studies conducted in Brazil^21^ and Vietnam^22^, as well as a study that used GYTS data from 140 countries^28^, and one that used data from six Sub-Saharan African countries^20^. In contrast, our study focused on both students who never smoked cigarettes and those who currently smoked cigarettes. Successful purchase of cigarettes among students who currently smoke cigarettes was high (65.4%), similar to findings from many other countries including a study that used GYTS data from 140 countries^28^. Studies conducted elsewhere including Ivory Coast (68.9%)^20^, South Africa (68.7%)^20^, Brazil (86.1)^21^, Vietnam (87.3% and 97.5% for boys and girls respectively)^22^, and China (80.5%)^23^ have shown that a high proportion of students were successful in purchasing cigarettes. A similar study in the US has also shown that only 24.3% of youths who attempted to purchase cigarettes were refused^32^. This suggests that children/youth purchase access to cigarettes is not only a challenge in The Gambia but also in many other countries.

Our findings have shown that the majority of students aged 11–17 years in The Gambia who currently smoked cigarettes bought them from a store, shop, street vendor, or canteen. Similarly, other studies found that shops and other retail outlets were the number one source of tobacco worldwide^28,33^. Prevention of access to cigarettes at retail outlets could be a good strategy to reduce smoking initiation and the prevalence of cigarette smoking. A study that used GYTS data from India, Pakistan, Bangladesh, and Sri Lanka showed that shopkeepers’ refusal to sell cigarettes to adolescents protected them from smoking^26^. As indicated in the 2016 Tobacco Control Act of The Gambia, any person who violates Section 17 (prohibition of supply of tobacco products to minors) of the Act commits an offence and is liable to a fine of ≥5000 GMD (about US$86 in 2023) or imprisonment of ≥3 months, or both, if convicted. The law also indicates that a fine of ≥100000 GMD (about US$1724 in 2023) will be imposed and the offender’s license will be suspended for ≥6 months, if the offender is a corporate entity^11^. Shopkeepers should be educated on the tobacco control laws that prohibit the sale of tobacco to and by minors, as well as the implications of selling cigarettes to children. There should be strong public health measures that target cigarette vendors to avoid selling cigarettes to minors.

Older age, higher school grade and current cigarette smoking were significantly associated with a successful attempt to purchase cigarettes. Surprisingly, higher weekly pocket money and parents’ smoking status were not significantly associated with a successful attempt to purchase cigarettes. This could be because 66.8% of students indicated neither of their parents’ smoke and hence it is possible that mainly other adult members of the students’ families and/or communities were sending them to purchase cigarettes. Parents and adults who smoke should be educated to desist from sending minors to purchase cigarettes or sharing cigarettes with them.

Some of the tobacco control laws in The Gambia were adopted (e.g. The Tobacco Control Act, 2016^13^, adopted in 2018) or passed (e.g. the Tobacco Control Regulations 2019^14^) after the survey took place in 2017. However, some of the provisions of the Tobacco Control Act, including those prohibiting sales to and by minors, tobacco advertising, promotion and sponsorship, and smoking in public places came into effect immediately in 2016. Both the current (2021–2025) and previous tobacco control policies (2013–2018) have provisions protecting children from access to tobacco^13^. In addition, the country signed and ratified the WHO FCTC including Article 16^10^, with a commitment to prevent the sale of tobacco to and by minors. This shows that The Gambia Ministry of Health and other stakeholders in tobacco control in the country already have the legal instruments that prohibit sale of tobacco to children^10,11,14^. There is evidence from a Cochrane Systematic Review suggesting enforcement of laws prohibiting cigarette sales to youths can lead to a reduction of smoking among youths^34^. In contrast, having laws without full implementation does not deter youths from accessing tobacco. For example, the 1993 Tobacco Products Control Act of South Africa prohibits the sale/supply of cigarettes to children aged <18 years, but the 2011 GYTS survey has shown that 68% of children aged 13–15 years who smoked cigarettes were successful in purchasing cigarettes^20^. Consistent compliance checks should be done to ensure vendors comply with the regulation. The age limit to purchase cigarettes should also be increased from 18 to 21 years and age verification conducted to avoid selling cigarettes to children.

Raising tobacco taxes, which will in turn increase the retail price, could be another effective strategy to prevent tobacco initiation among minors. As found in our previous study using the same GYTS data, increase in price deters children in The Gambia from experimenting with cigarette smoking^24^. Another study that used GYTS data from 16 African countries including The Gambia also revealed that higher cigarette prices decreased both the likelihood of smoking and smoking intensity among youth^35^.

### Limitations

Although the sample size was large, the study was conducted among students and therefore the findings may not be generalizable to children out of school. The study design was cross-sectional and hence did not allow causal analysis. Smoking behavior was self-reported, and students might have the tendency to under report or over report their smoking behavior. To understand how students who smoke obtain their cigarettes, we used the question: ‘The last time you smoked cigarettes during the past 30 days, how did you get them?’. Participants could pick only one response, which is a potential limitation as participants may have had access to all available options. Future surveys should ask where participants obtain products most frequently instead. Finally, the Tobacco Control Act 2016 was passed just one year before the survey. Therefore, this study cannot assess if the regulations have had a significant impact on the protection of children.

## CONCLUSIONS

We found that in 2017, children in The Gambia, including never smokers, were able to purchase cigarettes without much hinderance despite current regulations. To prevent tobacco initiation at a young age, the government should increase efforts in implementing the tobacco control laws that protect children. Our findings can guide the implementation of policies, programs, and public health strategies that can protect children from exposure and use of all forms of tobacco products.

## Data Availability

The data supporting this research are available from the following source: https://www.cdc.gov/tobacco/global/gtss/gtssdata/index.html.

## References

[1] World Health Organization (2022). Tobacco: Key facts.

[2] U.S. Department of Health and Human Services (2012). Preventing Tobacco Use Among Youth and Young Adults: A Report of the Surgeon General.

[3] Lynch BS, Bonnie RJ, Institute of Medicine (US) Committee on Preventing Nicotine Addiction in Children and Youths (1994). Growing up Tobacco Free: Preventing Nicotine Addiction in Children and Youths.

[4] Hair E, Bennett M, Williams V (2017). Progression to established patterns of cigarette smoking among young adults. Drug Alcohol Depend.

[5] National Center for Chronic Disease Prevention and Health Promotion (US) Office on Smoking and Health (2014). The Health Consequences of Smoking—50 Years of Progress: A Report of the Surgeon General.

[6] Jallow IK, Britton J, Langley T (2019). Prevalence and determinants of susceptibility to tobacco smoking among students in The Gambia. Nicotine Tob Res.

[7] van Loon AJ, Tijhuis M, Surtees PG, Ormel J (2005). Determinants of smoking status: cross-sectional data on smoking initiation and cessation. Eur J Public Health.

[8] Chen J, Millar WJ (1998). Age of smoking initiation: implications for quitting. Health Rep.

[9] World Health Organization (2003). WHO Framework Convention on Tobacco Control.

[10] World Health Organization WHO Framework Convention on Tobacco Control Implementation Database. Gambia: 2020 Report.

[11] Republic of The Gambia (2016). Tobacco Control Act, 2016.

[12] Republic of The Gambia Laws of the Gambia: Smoking (Prohibition in Public Places) Act 3 of 1998.

[13] Campaign for Tobacco-Free Kids Tobacco control laws: Legislation by country.

[14] Republic of The Gambia The Tobacco Control Regulations, 2019.

[15] Jallow IK, Britton J, Langley T (2017). Prevalence and determinants of tobacco use among young people in The Gambia. BMJ Glob Health.

[16] Manneh E (2008). A Global Youth Tobacco Survey (GYTS). Country Report (2008).

[17] Centers for Disease Control and Prevention (2017). Global Youth Tobacco Survey (GYTS) Factsheet.

[18] Cham B, Mdege ND, Bauld L, Britton J, D’Alessandro U (2021). Exposure to second-hand smoke in public places and barriers to the implementation of smoke-free regulations in The Gambia: a population-based survey. Int J Environ Res Public Health.

[19] Cham B, Scholes S, Groce NE, Mindell JS (2019). Prevalence and predictors of smoking among Gambian men: a cross-sectional National WHO STEP Survey. Int J Environ Res Public Health.

[20] Chandora R, Song Y, Chaussard M (2016). Youth access to cigarettes in six sub-Saharan African countries. Prev Med.

[21] Szklo AS, Cavalcante TM (2018). Noncompliance with the law prohibiting the sale of cigarettes to minors in Brazil: an inconvenient truth. J Bras Pneumol.

[22] Anh le TK, Quyen BT, Minh HV (2016). Tobacco access and availability for Vietnamese school children (aged 13-15): results from the Global Youth Tobacco Use Survey (GYTS) 2014 in Viet Nam. Asian Pac J Cancer Prev.

[23] Di X, Liu S, Xie H, Zeng X, Meng Z, Xiao L (2022). Cigarette availability and affordability among Chinese youth smokers: findings from the 2019 China Youth Tobacco Survey. Tob Induc Dis.

[24] Dare C, Cham B, Boachie MK, Gitonga Z, D’Alessandro U, Walbeek C (2022). Effect of price on the decision to experiment with cigarette smoking among Gambian children: a survival analysis using the Gambia 2017 Global Youth Tobacco Survey data. BMJ Open.

[25] Centers for Disease Control and Prevention Global Tobacco Surveillance System Data.

[26] Mishu MP, Siddiqui F, Shukla R, Kanaan M, Dogar O, Siddiqi K (2021). Predictors of cigarette smoking, smokeless tobacco consumption, and use of both forms in adolescents in South Asia: a secondary analysis of the Global Youth Tobacco Surveys. Nicotine Tob Res.

[27] Boachie MK, Immurana M, Tingum EN, Mdege ND, Ross H (2022). Effect of relative income price on smoking initiation among adolescents in Ghana: evidence from pseudo-longitudinal data. BMJ Open.

[28] Sun J, Xi B, Ma C, Li Z, Zhao M, Bovet P (2022). Cigarette access and purchase patterns among adolescent smokers aged 12-16 years in 140 countries/territories, Global Youth Tobacco Survey 2010-2018. J Glob Health.

[29] Global Youth Tobacco Survey Collaborative Group (2014). Global Youth Tobacco Survey (GYTS): Core Questionnaire with Optional Questions, Version 1.2.

[30] Portnoy DB, Wu CC, Tworek C, Chen J, Borek N (2014). Youth curiosity about cigarettes, smokeless tobacco, and cigars: prevalence and associations with advertising. Am J Prev Med.

[31] Nodora J, Hartman SJ, Strong DR (2014). Curiosity predicts smoking experimentation independent of susceptibility in a US national sample. Addict Behav.

[32] Tanski S, Emond J, Stanton C (2019). Youth access to tobacco products in the United States: findings from Wave 1 (2013-2014) of the Population Assessment of Tobacco and Health Study. Nicotine Tob Res.

[33] D’Angelo D, Ahluwalia IB, Pun E, Yin S, Palipudi K, Mbulo L (2016). Current cigarette smoking, access, and purchases from retail outlets among students aged 13-15 Years - Global Youth Tobacco Survey, 45 Countries, 2013 and 2014. MMWR Morb Mortal Wkly Rep.

[34] Stead LF, Lancaster T (2005). Interventions for preventing tobacco sales to minors. Cochrane Database Syst Rev.

[35] Filby S, van Walbeek C (2022). Cigarette prices and smoking among youth in 16 African countries: evidence from the Global Youth Tobacco Survey. Nicotine Tob Res.

[36] Central Bank of The Gambia Weekly Valuation Exchange Rates 2023.

